# Elevated expression of minichromosome maintenance 3 indicates poor outcomes and promotes G1/S cell cycle progression, proliferation, migration and invasion in colorectal cancer

**DOI:** 10.1042/BSR20201503

**Published:** 2020-07-09

**Authors:** He Zhou, Yongfu Xiong, Guangjun Zhang, Zuoliang Liu, Lifa Li, Songlin Hou, Tong Zhou

**Affiliations:** 1 The Second Department of Gastrointestinal Surgery, Affiliated Hospital of North Sichuan Medical College, Nanchong 637000, Sichuan Province, China; 2 The First Department of Hepatobiliary Surgery, Affiliated Hospital of North Sichuan Medical College, Nanchong 637000, Sichuan Province, China; 3 Institute of Hepatobiliary, Pancreatic and Intestinal Disease, North Sichuan Medical College, Nanchong 637000, Sichuan Province, China

**Keywords:** colorectal cancer, MCM3, minichromosome maintenance

## Abstract

**Background:** The minichromosome maintenance (MCM) family, a core component of DNA replication, is involved in cell cycle process. Abnormal proliferation has been identified as a crucial process in the evolution of colorectal cancer (CRC). However, the roles of the MCM family in CRC remain largely unknown.

**Methods:** Here, the expression, prognostic significance and functions of the MCM family in CRC were systematically analyzed through a series of online databases including CCLE, Oncomine, HPA, cBioPortal and cancerSEA.

**Results:** We found all MCM family members were highly expressed in CRC, but only elevation of MCM3 expression was associated with poor prognosis of patients with CRC. Further *in vitro* and *in vivo* experiments were performed to examine the role of MCM3 in CRC. Analysis of CCLE database and qRT-PCR assay confirmed that MCM3 was overexpressed in CRC cell lines. Moreover, knockdown of MCM3 significantly suppressed transition of G1 to S phase in CRC cells. Furthermore, down-regulation of MCM3 inhibited CRC cell proliferation, migration, invasion and promoted apoptosis.

**Conclusion:** These findings reveal that MCM3 may function as an oncogene and a potential prognosis biomarker. Thus, the association between abnormal expression of MCM3 and the initiation of CRC deserves further exploration.

## Introduction

Colorectal cancer (CRC) is the third most prevalent malignant cancer globally. Besides fast progression, CRC is associated with high mortality [1]. In 2019, approximately 1,762,450 and 606,880 new cases and deaths reported in the United States of America, respectively [2]. Although the advent of more accurate early screening methods and personalized treatments has stabilized the number of CRC cases in developed countries, the incidence and mortality in developing countries, including China, continue to rise significantly [3,4]. Currently, the TNM staging method is based on the American Joint Committee on Cancer (AJCC) and the Union for International Cancer Control (UICC). It is used to assess the prognosis of patients and guide the choice of treatment. However, given that treatment outcomes of individual patients are unpredictable [5], in-depth understanding of the underlying molecular mechanisms and identification of CRC markers are required to develop effective therapeutic strategies.

Uncontrolled self-replication of tumor cells promotes cancer progression, and refers not only to abnormal proliferation at the primary site but also to rapid growth in the target organs for metastasis [6]. Cell proliferation is regulated by multiple mechanisms, with DNA replication being a crucial regulator that ensures precise control of the cell cycle [7]. The minichromosome maintenance family (MCMs) was first identified in *Saccharomyces cerevisiae*, where its mutation was shown to weaken the stability of minichromosome in proliferating cells, thereby stalling continuity of the cell cycle [8,9]. So far, nine homologues of MCM proteins have been identified in eukaryotic cells including MCM2-7, MCM8, MCM9 and MCM10 [10–13]. These proteins are involved in the initiation of DNA replication [14], transcription [15], cycle checkpoint [16] and RNA splicing [17]. Among them, MCM2-7 recruits ORC, CDC6 and CDT1 to form heteromultimers, which function as helicases driving the transition from M to G1 phase [18]. Although similar to MCM2-7 helicase in terms of the replication process, the proteins involved in loading MCM8 and MCM9 are different [19]. Recent studies have indicated that during homologous recombination, MCM8 and MCM9 can stabilize each other and form a complex that promotes DNA synthesis thereby reactivating a broken replication fork [20]. MCM10 is highly conserved in eukaryotic cells, and promotes continuous replication of the cell cycle by recruiting DCAF1-Cul4-E3 [21].

Accumulating evidence indicates that DNA replication-related proteins can be novel therapeutic targets for tumors [22]. Due to their close association with the cell replication process, the MCM family proteins are considered ideal biomarkers for malignant cell proliferation. Besides, abnormal expression of MCMs has been detected in a variety of tumors, including breast cancer [23], hepatocellular carcinomas [24], renal cancer [25], colorectal cancer [26] and gliomas [27]. Furthermore, evidence has emerged that associates abnormal expression of MCMs with cancer prognosis. For instance, MCM2 is a promising prognostic marker in lung squamous cell carcinoma, where its overexpression indicates a shorter survival period [28]. High expression level of MCM5 correlates with tumor invasiveness and unfavorable prognosis of cervical cancer [29]. MCM4 influences the cell cycle in colorectal cancer by regulating (Skp2)-P27 axis and Ohmyungsamycin A expression [30]. High expression of MCM7 promotes cell proliferation by activating the MAPK signaling pathway, leading to worse prognosis in hepatocellular carcinoma [31]. The depletion of MCM9 can lead to an early susceptibility to colorectal cancer [32].

In the MCM2–7 complex, MCM3 functions as a flexible linker through its C-terminal [33]. Unlike cell cycle biomarkers ki-67 and proliferating cell nuclear antigen (PCNA), MCM3 possesses higher stability and is less susceptible to interference from external factors [34]. Although several lines of evidence indicate that MCMs play a key role in tumor progression, but their prognostic value in CRC remain unknown. In the present study, we aimed to screen for valuable prognostic and diagnostic markers to imporve the treatment of CRC by conducting an integrated analysis of several databases based on the mRNA expression profile of the MCM family.

## Materials and methods

### Oncomine database analysis

Oncomine (http://www.oncomine.org) is currently the world’s largest oncogene chips database and integrated data-mining platform for cancer genetic information. It covers 65 microarray data set, 4700 chips, and 480 million gene expression data. Oncomine is used for analysis of gene expression differences, identification of outliers, predicting co-expressed genes, and facilitating the discovery of new tumor biomarkers, or therapeutic targets [35]. Further, it integrates TCGA and GEO data as well as some bioinformatics analysis tools for standardized analysis of tumor transcriptome data. In the present study, we used the database to analyze the transcription levels of distinct MCMs in tumor and normal tissues for different types of cancers. The results were filtered using the threshold: fold change ≥ 2; *P*-value ≤ 1 × 10^−4^; gene rank ≥ top 10%.

### The human protein atlas database

The Human Protein Atlas (HPA) is a large database containing tissue and cell distribution information for 24,000 proteins in humans. These include the immunohistochemical (IHC) test results showing the distribution and expression of each protein in 48 human normal tissues, 20 tumor tissues, 47 cell lines, and 12 blood cells, which have been validated and indexed by experts [36]. It also includes the possible roles of specific proteins in the development of different tumors, making it an platform for tumor research. Therefore, IHC images of cancer and normal tissues in patients with CRC were obtained from the HPA database, and the protein expression of MCMs was visualized.

### Analysis of cBioPortal database

With the widespread application of chip and high-throughput sequencing technology, massive genomic data have been generated in the field of oncology, and a variety of databases have been designed to meet different research needs. The cBioPortal (http://www.cbioportal.org/) database integrates data from 126 tumor genomic studies, including large-scale tumor research projects such as TCGA and Oncomine, and contains data from 28,000 specimens [37]. In addition, some samples also include information such as clinical prognosis. This database provides researchers with high-quality molecular profiling, and clinical prognostic correlation of large-scale cancer genomics projects, within a short time [38]. In our study, we analyzed the relationship between clinical prognosis and MCMs in patients with CRC using the database.

### CancerSEA database analysis

CancerSEA (http://biocc.hrbmu.edu.cn/CancerSEA/) is the first single-cell sequencing database designed to comprehensively explore the functional states of cancer cells at single-cell level. The single-cell sequencing results in the CancerSEA database are derived from 72 data sets in the SRA, GEO and ArrayExpress websites. In total, it comprises 41,900 cancer cells from 25 types of cancer, and functional analysis results from data sets such as HCMDB, Cyclebase and StemMapper, which have redefined 14 functional states [39]. Therefore, we used the CancerSEA database to analyze the functional correlation of the MCM family with CRC.

### Cancer cell line encyclopedia database analysis

The Cancer cell line encyclopedia (CCLE) (https://portals.broadinstitute.org/ccle) contains sequencing information of 947 human cancer cell lines from more than 30 tissue sources, and includes data visualization [40]. To deepen our understanding of DNA mutations, gene expression and chromosome copy number information of specific genes, we used CCLE to analyze the mRNA expression of MCM family members in cell lines derived from different tumor types. The raw microarray data of MCMs in CRC cell lines were downloaded from CCLE and then converted to a single value for each probe set using the RMA algorithm and quantile normalization.

### Cell culture

The normal human colon cell line NCM460 and human CRC cell line COLO205 were obtained from the Chinese Academy of Sciences Cell Bank. All the cells were cultured in roswell park memorial institute (RPMI)-1640 medium containing 10% fetal bovine serum (PAN-Biotech, Adenbach, Bavaria) in a 37°C constant temperature incubator with 5% CO_2_.

### Lentivirus transfection and stable cell line selection

The lentivirus-mediated GV248 vector (Genechem, China) was used to express short hairpin RNA (shRNA) targeted MCM3. The sequences of shRNAs targeting MCM3 were as follows: 5′-CCA GAG GTA GAC GTA TTT ATT-3′ (sense) and 5′-GCC TGG CTA GGG TTA GGA CTT-3′ (antisense). Lentiviral particles were transfected into 293T cells with GV248-shMCM3 constructs. The stable cell lines were selected by puromycin (4 μg/ml) 72 h after transfection. The medium containing puromycin was replaced every 3 days for 2 weeks.

### RNA isolation and quantitative real-time PCR

TRIzol reagent (Invitrogen, U.S.A.) was used to extract total RNA from cells. The extracted RNA was reverse-transcribed using the first strand cDNA synthesis kit (Thermo, #K1642). Then, qRT-PCR was carried out on a Bio-Rad CFX96 system (Bio-Rad, Hercules, CA). Primer sequences in the present study were: 5′-CGA GAC CTA GAA AAT GGC AGC C-3′ (forward) and 5′- GCA GTG CAA AGC ACA TAC CGC A-3′ (reverse) for MCM3; 5′-GTC TCC TCT GAC TTC AAC AGC G-3′ (forward) and 5′-ACC ACC CTG TTG CTG TAG CCA A-3′ (reverse) for GAPDH. GAPDH was used as a normalizing control and the relative mRNA expression was calculated using the ΔΔCT method.

### Western blotting analysis

Cells were harvested at a density above 90% and subsequently lysed in RIPA buffer (Solarbio, China) on ice. Equal protein lysates (20 μg) were transferred to polyvinylidene fluoride membranes (Millipore, County Cork, Ireland) through sodium dodecyl sulfate polyacrylamide gels. Membranes were blocked in rapid block buffer (Sangon Biotech, China) for 15 min and then incubated at 4°C overnight with relevant primary antibodies. The Horse Radish Peroxidase-conjugated secondary antibodies were then used to incubate the membranes for 1 h at room temperature. Primary antibodies used were rabbit anti-MCM3 (dilution, 1:2000; cat. no., ab80044; Abcam), rabbit anti-CDK2 (dilution, 1:1000; cat. no., 2546; Cell Signaling), rabbit anti-CDK4 (dilution, 1:1000; cat. no., 12790; Cell Signaling), rabbit anti-Cyclin D1 (dilution, 1:1000; cat. no., 26939-1-AP; Proteintech), rabbit anti-Cyclin E1 (dilution, 1:1000; cat. no., 11554-1-AP; Proteintech), and rabbit anti-β-actin (dilution, 1:5000, cat. no., 20536-1-AP; Proteintech).

### Cell apoptosis and cycle analysis

Cell apoptosis and cycle were detected by flow cytometry (FCM). Briefly, adherent cells were separated into single cells using 0.25% trypsin; the cell suspensions were mixed with PBS and centrifuged for 5 min at 1000 r/min. The supernatant was discarded and 1 × 10^6^ cells were resuspended in 500 μl of PBS and incubated in AnnexinV-APC and DAPI in the dark for 20 min. A cell suspension containing 1 × 10^6^ cells was stained with propidium iodide (PI) and analyzed by FCM for cell cycle distribution.

### Cell colony formation assay

Colony formation assay was conducted to evaluate the effect of MCM3 knockdown on cell proliferation. Transfected cells (shMCM3 or shCtrl) were seeded in six-well plates at a density of 500 per well; three replicate wells were set for each group. About 2 ml of RPMI-1640 medium containing 10% FBS was added into each well and cultured for 2 weeks. The formed clones were washed twice with PBS, fixed with methanol for 20 min and subsequently stained with 0.2% Crystal Violet for 10 min. A cell colony with more than 50 cells was considered a positive colony and the number of colonies were counted under a microscope.

### Cell migration and invasion assay

The migration and invasion ability of cells was determined using wound healing and transwell assays. For the wound healing assay, 5 × 10^5^ cells were seeded in a 6-well plate and allowed to grow until a confluence of 95%. An artificial wound was created with a 10 μl sterile tip. Cells were incubated a serum-free medium for 24 h, after which the wound was imaged under a microscope. Cell migration was assessed by measuring the spacing between the two boundaries of the cell area. For cell transwell assay, 100 μl of serum-free medium containing 1 × 10^5^ cells was plated in the upper chamber whereas a medium containing 10% FBS was added to the lower chamber and cultured for 24 h under standard conditions. The cells that had migrated into the lower chamber were fixed with 4% polymethanol for 20 min and stained with 0.2% Crystal Violet for 5 min. Invaded cells were observed under an inverted microscope and counted in five random fields of view.

### Xenograft *in vivo* analysis

Ten BALB/c nude mice were purchased from the Shanghai Laboratory Animal Center (Shanghai, China). All animal studies were conducted in full compliance with the principles and protocols approved by the Ethics Committee on Animal Center of North Sichuan Medical College. After the mice grew to 4 weeks old, they were administered 2% cocaine hydrochloride anesthesia on the skin surface. To evaluate the tumorigenicity of stable knockdown of MCM3 cells in vivo, CRC cells were transfected with lentivirus (COLO205-shMCM3) to knockdown the expression of MCM3 or with COLO205-shCtrl. About 100 μl of the cell suspension (1 × 10^7^ cells/ml) was injected into the lateral abdomen of nude mice (*n* = 5 per group). The survival status of mice was observed and their tumor size recorded every 3 days. Four weeks after the injection, mice were killed by cervical dislocation. After their heart and breathing had completely stopped, the formed neoplasms were excised and weighed for immunohistochemical assay.

### Immunohistochemistry staining

Excised tumor tissues were continuously sliced into 5-μm sections. The slides were dewaxed in xylene and dehydrated with a series of ethanol concentrations. Antigen retrieval was performed by treating the tissue sections with citrate buffer and heating them a microwave. The tissues were incubated with primary mouse monoclonal antibody against PCNA (dilution, 1:500; cat. no., 60097-1-Ig; Proteintech) overnight at 4°C. Next, they were incubated with HRP-labeled secondary antibody (Santa cruz, sc-516102) for 30 min and stained with the DAB immunohistochemistry color development kit (Sangon Biotech, China). Finally, the dyed tissue sections were examined under a microscope (Olympus, Japan).

### Statistical analysis

The GraphPad Prism (Version 8.0 GraphPad Software, CA) was used for statistical analyses. The significance of differences between groups was evaluated using the Student’s *t*-test. Statistical significance of MCM family expression between colorectal cancer and normal tissues from Oncomine was provided by the program. Survival data of MCM family mRNA expression were obtained from cBioPortal. Survival curves were plotted via the Kaplan–Meier method and compared with the log- rank test. *P* values less than 0.05 were considered statistically significant.

## Results

### mRNA expression profile of MCM family members in CRC

**Figure 1:**
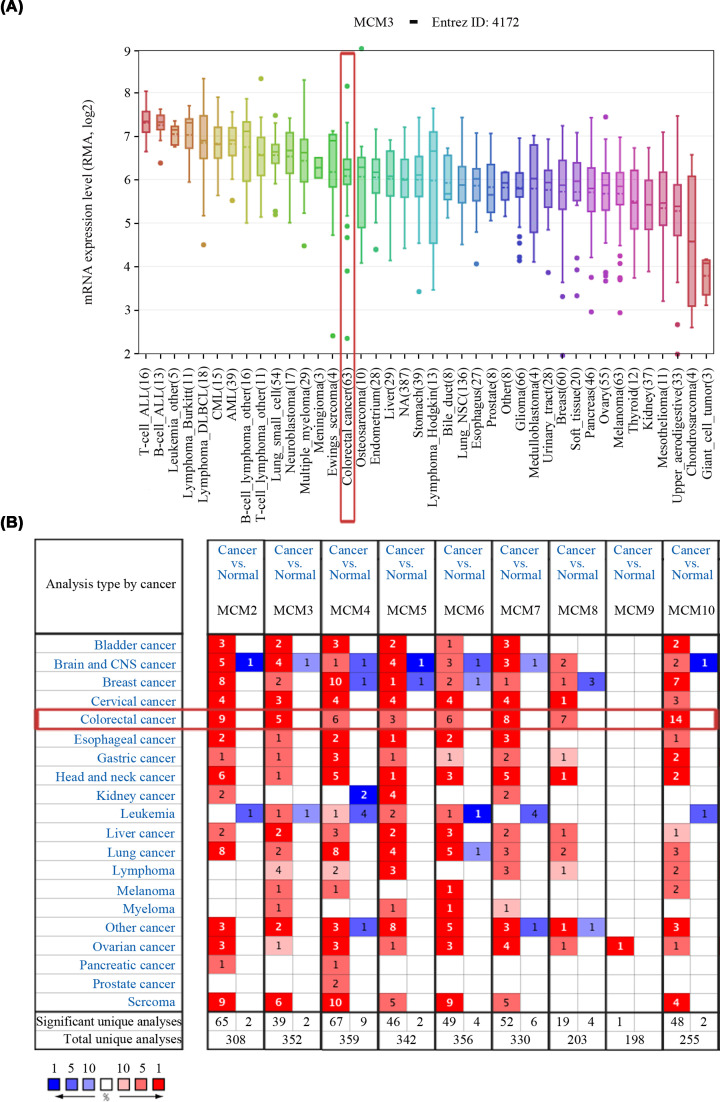
mRNA expression of MCM family members in various types of cancer (**A**) The mRNA expression level of MCM3 in CCLE database. MCM3 mRNA expression level ranks 15th among a group of human cancers (shown in red frame). (**B**) The mRNA expression levels of MCM family members in various types of cancer versus normal tissues in the Oncomine database. The blue box in the graph indicates that the target gene is down-regulated in the corresponding tumor, while red indicates up-regulated genes, with statistically significant differences (*P* = 1 × 10^−4^). The number in the cell represents the number of studies that meet the set threshold. The color of the cells is determined by the rank of gene expression differences; CCLE, cancer cell line encyclopedia.

CCLE analysis revealed that the expression of MCM3 in colorectal cancer ranked 15th among all types of cancer (Figure 1A). Analysis performed on the Oncomine database showed that the mRNA expression of all MCM family members was overexpressed in CRC compared with normal tissues across diversified datasets (Figure 1B).

**Figure 2:**
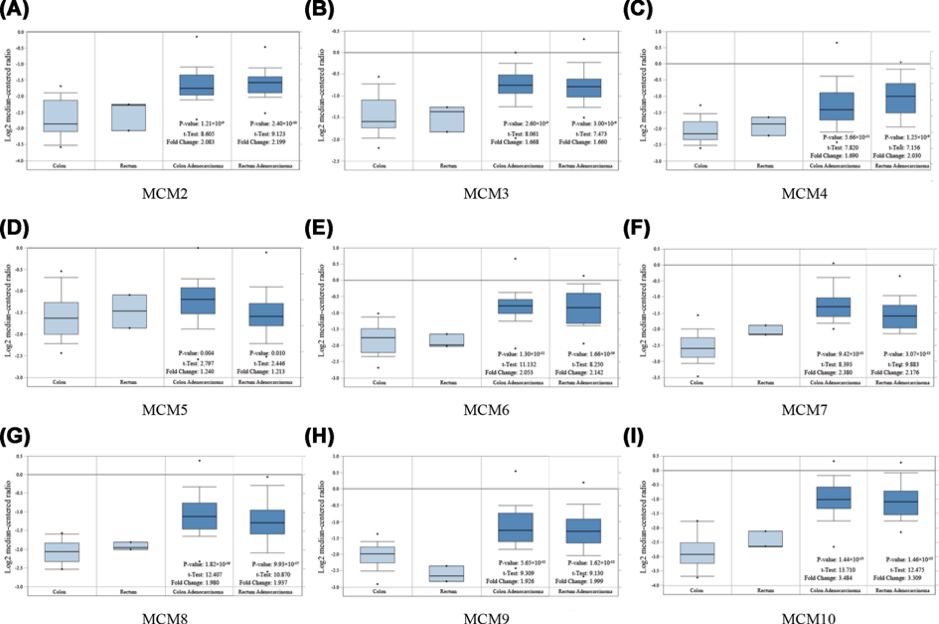
Expression pattern of MCM family members in colorectal cancer and normal tissues (Oncomine database) Box plots derived from gene expression data comparing expression levels of a specific MCM family member in CRC and corresponding normal tissue. The *P* value was set up at 1 × 10^−4^ and fold change was defined as two. (**A**) Analysis of MCM2 mRNA expression. (**B**) Analysis of MCM3 mRNA expression. (**C**) Analysis of MCM4 mRNA expression. (**D**) Analysis of MCM5 mRNA expression. (**E**) Analysis of MCM6 mRNA expression. (**F**) Analysis of MCM7 mRNA expression. (**G**) Analysis of MCM8 mRNA expression. (**H**) Analysis of MCM9 mRNA expression. (**I**) Analysis of MCM10 mRNA expression; CRC, colorectal cancer. *P<0.05.

MCM mRNA expression in CRC and normal tissues were summarized in Table 1. In a TCGA dataset with the largest sample size (*n*=237) from the Oncomine database, MCM3 transcripts in the colon and rectal adenocarcinoma tissues were 1.668- and 1.660-fold higher than relevant normal tissues respectively (Figure 2B). Moreover, MCM2 was 2.083- and 2.199-fold higher in colon and rectal adenocarcinoma samples respectively compared with normal tissues (Figure 2A). The MCM4 was 1.690- and 2.030-fold as high as the normal tissues in colon and rectal adenocarcinoma samples, respectively (Figure 2C). MCM5 was higher 1.240-fold in colon and 1.213-fold in rectal adenocarcinoma samples than in normal tissues (Figure 2D). The MCM6 was 2.053- and 2.142-fold higher in colon and rectal adenocarcinoma samples respectively compared with the normal tissues (Figure 2E). MCM7 was 2.380- and 2.176-fold higher in the colon and rectal adenocarcinoma samples in that order compared with contrastingly relevant normal tissues (Figure 2F). Furthermore, MCM8 was found to be 1.980- and 1.937-fold higher in colon and rectal adenocarcinoma samples respectively as compared with relevant normal tissues (Figure 2G). MCM9 was 1.926-fold in colon and 1.999-fold in rectal adenocarcinoma samples lower than relevant normal tissues (Figure 2H). MCM10 was also 3.484-fold in colon and 3.309-fold in rectal adenocarcinoma samples lower than relevant normal tissues (Figure 2I).

**Table 1:** Comparison of mRNA expression of MCMs in colorectal cancer and normal tissues from the Oncomine database

**Gene**	MCM2	MCM3	MCM4	MCM5	MCM6	MCM7	MCM8	MCM9	MCM10
**Case, ***n*****									
Colon	19	19	19	19	19	19	19	19	19
Rectal	3	3	3	3	3	3	3	3	3
Colon cancer	101	101	101	101	101	101	101	101	101
Rectal cancer	60	60	60	60	60	60	60	60	60
**Detection type**	mRNA	mRNA	mRNA	mRNA	mRNA	mRNA	mRNA	mRNA	mRNA
**Fold change**									
Colon cancer vs. Normal	2.083	1.668	1.690	1.240	2.053	2.380	1.980	1.926	3.484
Rectal cancer vs. Normal	2.199	1.660	2.030	1.213	2.142	2.176	1.937	1.999	3.309
***T*-test**									
Colon cancer vs. Normal	8.605	8.061	7.820	2.797	11.132	8.393	12.407	9.309	13.710
Rectal cancer vs. Normal	9.123	7.473	7.156	2.446	8.250	9.883	10.870	9.130	12.475
***P*-value**									
Colon cancer vs. Normal	1.21 × 10−9	2.60 × 10−9	5.66 × 10−11	0.004	1.30 × 10−12	9.42 × 10−11	1.82 × 10−18	5.65 × 10−12	1.44 × 10−15
Rectal cancer vs. Normal	2.40 × 10−10	3.00 × 10−9	1.25 × 10−9	0.010	1.66 × 10−10	3.07 × 10−12	9.93 × 10−17	1.62 × 10−12	1.46 × 10−15
**TCGA, ID**	A_23_P250808	NM_002388_2_2539	X74794_1_2840	A_23_P132279	NKI_NM_005915	A_23_P93690	A_23_P68547	A_23_P397347	A_23_P161474

### Protein expression of MCMs in CRC tissues

**Figure 3:**
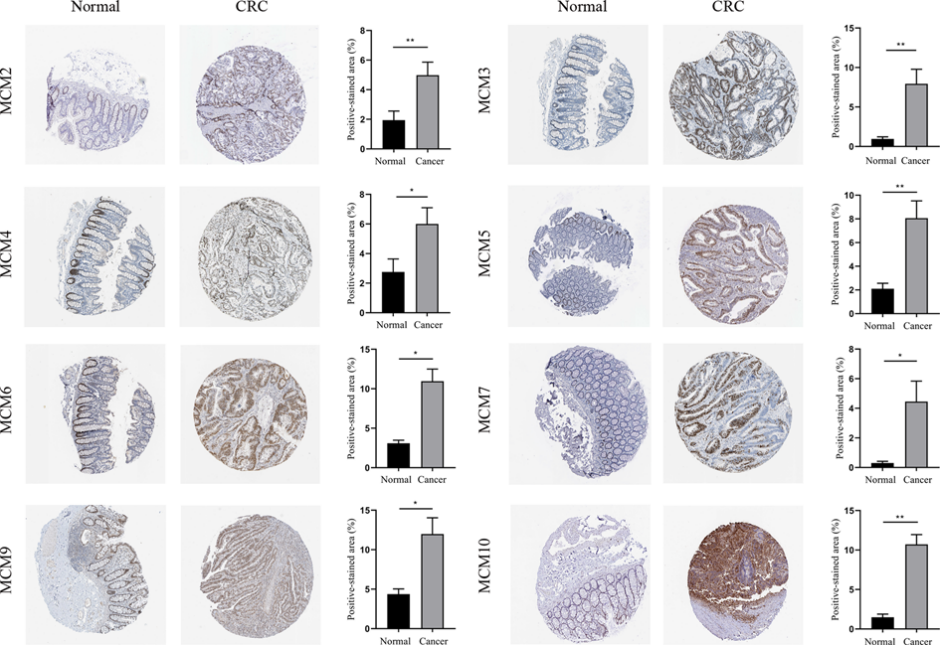
Immunohistochemical analysis of protein expression in CRC tissues and normal tissues (The Human Protein Atlas Database). The brown areas represent positive expression, the blue areas represent negative expression CRC, colorectal cancer. *P<0.05, **P<0.01.

The expression of MCMs in CRC tissues was analyzed using MCMs immunohistochemical results obtained from Human Protein Atlas database (Figure 3). We found that protein levels of all MCM members were higher in CRC tissues than in normal tissues. These results were consistent with those seen in mRNA expression.

### The impact of MCM family expression on prognosis of CRC patients

**Figure 4:**
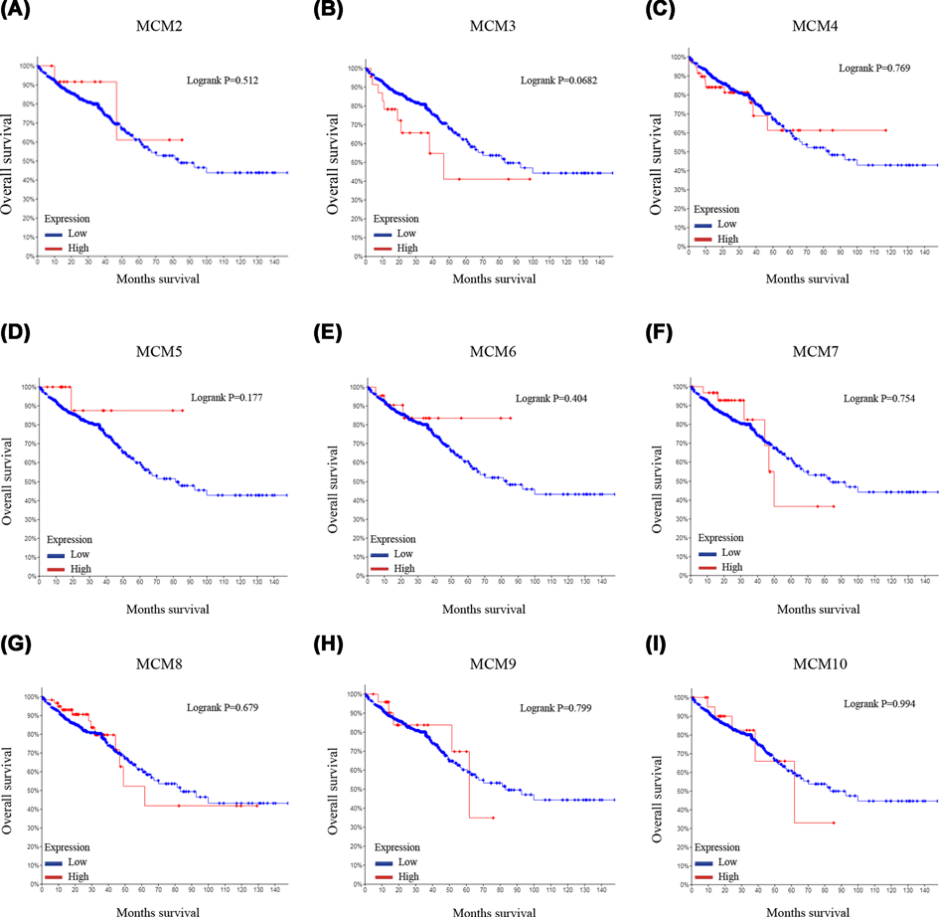
Kaplan–Meier survival analysis for OS of CRC patients based on MCM family mRNA expression (**A–I**) OS curves of MCM2, MCM3, MCM4, MCM5, MCM6, MCM7, MCM8, MCM9 and MCM10 in patients with CRC; CRC, colorectal cancer; OS, overall survival.

**Figure 5:**
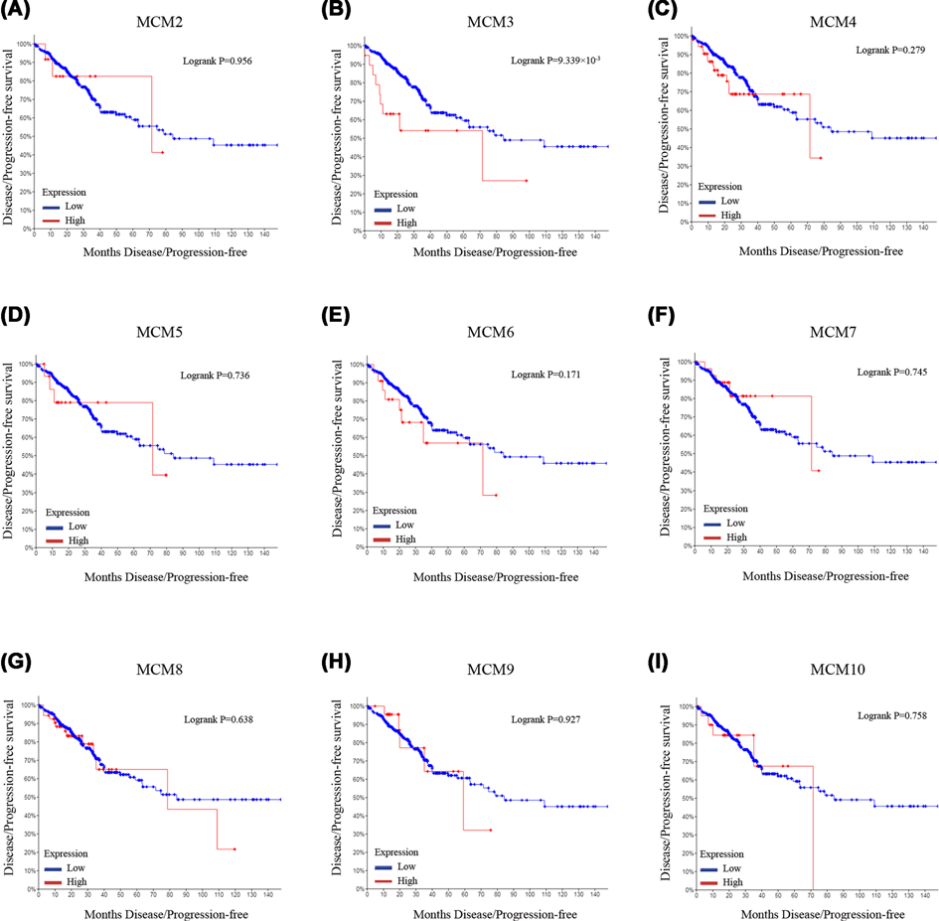
Kaplan–Meier survival analysis for DFS of CRC patients based on MCM family mRNA expression (**A–I**) DFS curves of MCM2, MCM3, MCM4, MCM5, MCM6, MCM7, MCM8, MCM9 and MCM10 in patients with CRC. DFS, disease-free survival; CRC, colorectal cancer.

Further analysis of the prognostic implication of mRNA levels of MCM family in CRC performed in cBioPortal database revealed that patients with high MCM3 expression had worse DFS/PFS outcome compared with those with low MCM3 expression (*P*=0.009339, Figure 5B). However, mRNA levels of the remaining MCM family members were not significantly correlated with OS or DFS/PFS (*P*>0.05, Figures 4 and 5A,C–I).

### The functions of MCM family members in a single CRC cell

**Figure 6:**
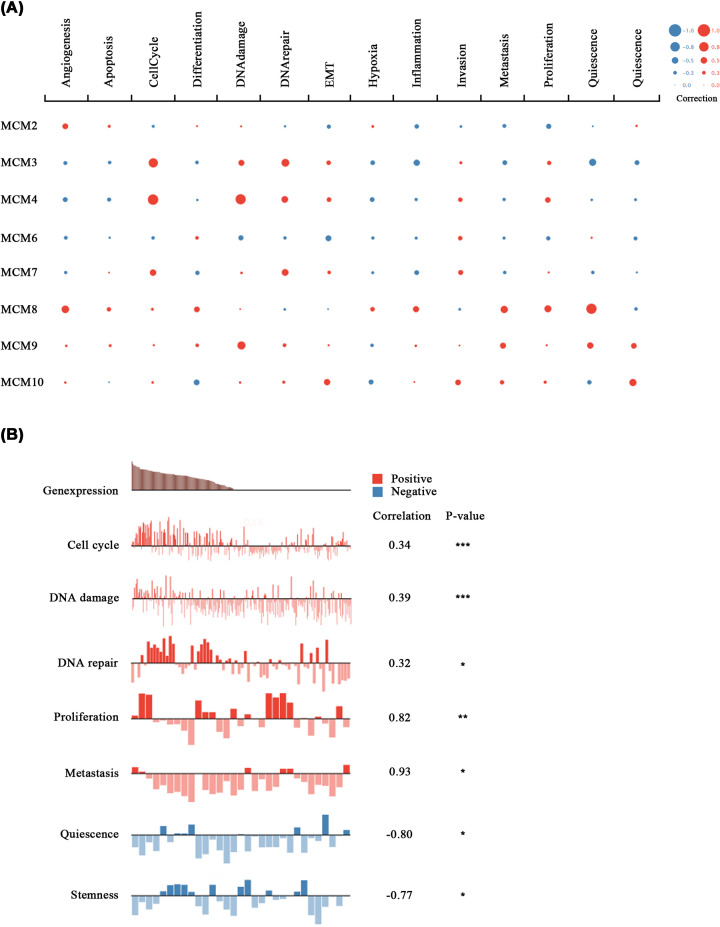
Functional analysis of MCM family members in CRC using CancerSEA database (**A**) Functional relevance of each MCM member in CRC cells. The size of the bubble indicates the strength of the correlation; red represents a positive correlation and blue represents a negative correlation. (**B**) Details of the functional relevance of MCMs in CRC. *P<0.05, **P<0.01 and ***P<0.001; CRC, colorectal cancer.

Heterogeneity associated with different functional phenotypes of tumor cells has been major obstacle to effective treatment of cancer. Recent advances in single-cell sequencing (scRNA-seq) technology have provided a tool for understanding the functional status of tumor cells at the cellular level. Functional correlation analysis of cancerSEA showed that the functional phenotypes of MCM family in CRC cells were positively correlated with cell cycle, DNA damage, DNA repair, proliferation and metastasis, but negatively correlated with quiescence and stemness (Figure 6B). In addition, MCM3 played a role in cell cycle, invasion and proliferation in CRC cells (Figure 6A).

### The mRNA expression of MCM3 in CRC cell lines

**Figure 7:**
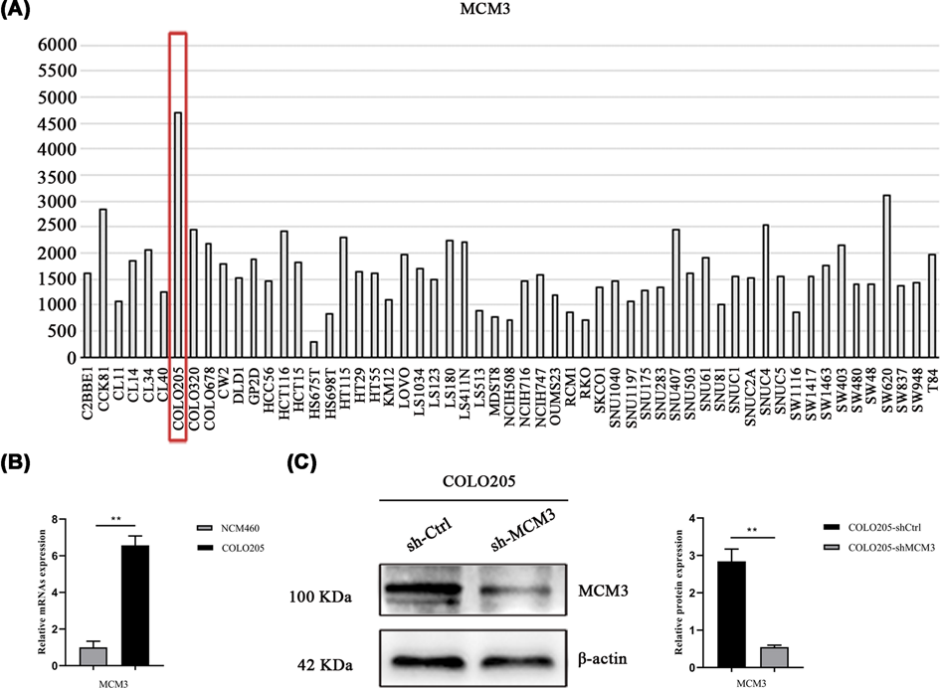
MCM3 is up-regulated in CRC cell lines (**A**) The mRNA level of MCM3 in different CRC cell lines from CCLE analysis. (**B**) The mRNA expression of MCM3 in COLO205 and normal colon mucosal epithelial cell was detected by qRT-PCR (*n*=3 independent experiments **P<0.01). (**C**) The protein levels of MCM3 in stably-transduced sh-Ctrl and sh-MCM3 COLO205 cell line were analyzed by Western blot (*n*=3 independent experiments **P<0.01); CCLE, cancer cell line encyclopedia; CRC, colorectal cancer; qRT-PCR, quantitative real-time polymerase chain reaction.

Having shown that MCM3 expression in COLO205 cell was higher than in other CRC cell lines based on CCLE analysis (Figure 7A), we used COLO205 cells in the subsequent analysis. Thus, qRT-PCR assay was performed to determine the mRNA levels of MCM3 in CRC cell lines and normal cell lines. As shown in Figure 7B, the mRNA expression of MCM3 was higher in the COLO205 cell line compared with the normal cell line NCM460.

### Effect of MCM3 knockdown on cell cycle and apoptosis

**Figure 8:**
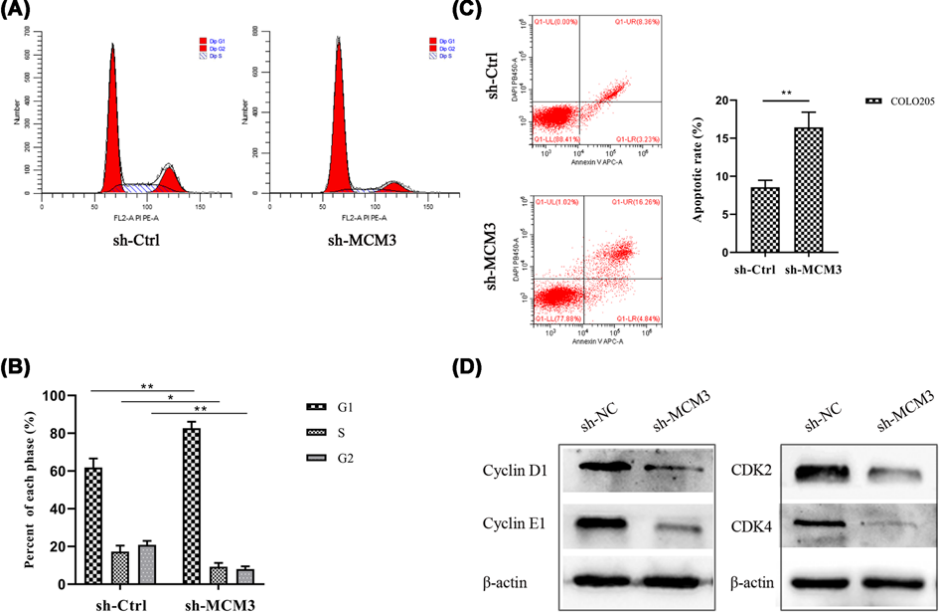
Effect of MCM3 knockdown on CRC cell cycle distribution and apoptosis (**A**) The cell cycle distribution of COLO205 cell was detected by FCM. (**B**) The results of FCM measurement showed that the cell cycle was arrested in G1 phase after the MCM3 knockdown. (**C**) Apoptosis ratios of MCM3 in stably transduced sh-Ctrl and sh-MCM3 COLO205 cells were detected by FCM analysis. (**D**) The protein expression of CDK2, CDK4, CyclinD1 and CyclinE1 was significantly inhibited after MCM3 knockdown; CRC, colorectal cancer; FCM, flow cytometry. All representative data are from three independent experiments; *P<0.05, **P<0.01.

Accumulating evidence has confirmed that members of the MCM family play a key role in DNA replication. Our previous analysis uncovered that MCMs are interlinked with cell cycle-related genes. Therefore, we investigated the effect of MCM3 knockdown on the cell cycle of CRC cells. Results shown in Figure 7C indicate that the protein levels of MCM3 were significantly reduced after MCM3 knockdown. The proportion of cells in the G1 phase was higher, while the proportion of cells in the S phase was significantly lower in sh-MCM3 group than in the sh-Ctrl group (Figure 8A,B). On the other hand, apoptosis was significantly higher in cells expressing sh-MCM3 compared with cells of sh-Ctrl group (Figure 8C). Mechanistically, MCM3 knockdown significantly inhibited the expression of proteins associated with cell cycle initiation such as CDK2, CDK4, CyclinD1 and CyclinE1 (Figure 8D). These results suggested that MCM3 interferes with cell cycle progression causing abnormal proliferation of CRC cells.

### MCM3 knockdown inhibits CRC cells proliferation, migration, invasion and tumor growth *in vitro* and *in vivo*

**Figure 9:**
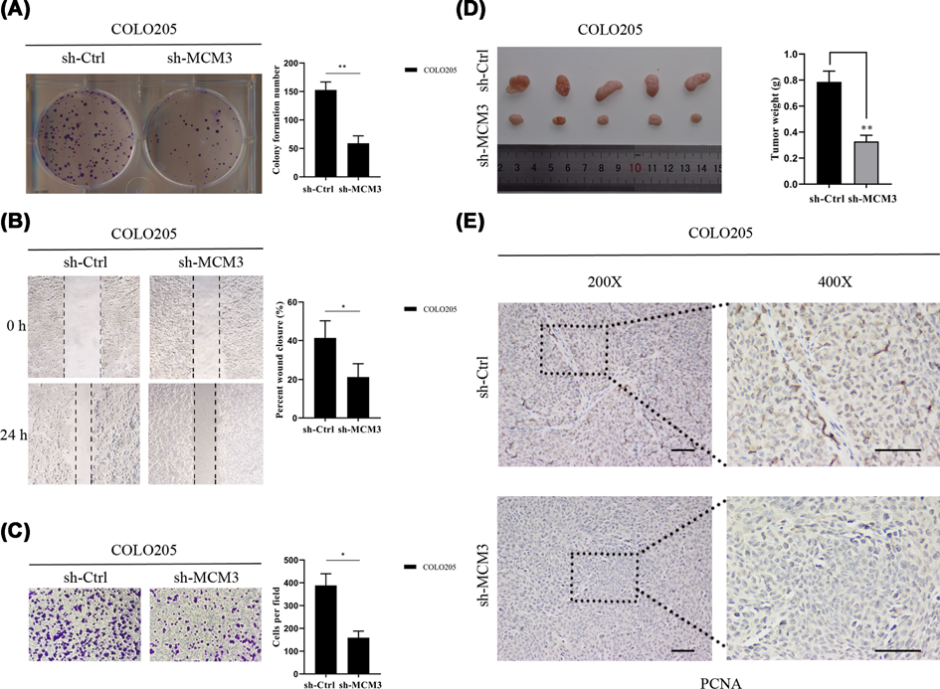
Knockdown of MCM3 inhibits CRC cell proliferation, migration and invasion *in vitro* and *in vivo* (**A**) Knockdown of MCM3 suppressed cell proliferation as indicated by colony formation assays in COLO205 cells (*n*=3 independent experiments **P<0.01). (**B**) Knockdown of MCM3 significantly inhibited cell migration in COLO205 cells (*n*=3 independent experiments, *P<0.05). (**C**) Knockdown of MCM3 significantly inhibited cell invasion in COLO205 cells (*n*=3 independent experiments, *P<0.05). (**D**) Knockdown of MCM3 markedly inhibited tumor growth of COLO205 cells *in vivo*. Images of tumors excised from the mice were presented and the tumor weight data was shown as the mean ± SD; **P<0.01. (**E**) Images of IHC staining of PCNA in xenograft tumors; scale bar, 50 μm; CRC, colorectal cancer; IHC, immunohistochemical; PCNA, proliferating cell nuclear antigen.

To determine the biological functions of MCM3 in CRC, MCM3 was knocked down in CRC cells. A colony formation assay performed on the cells indicated that MCM3 silencing decreased the number of colonies in formed by COLO205 cells (*P*<0.05, Figure 9A). Furthermore, wound healing and transwell assays revealed a significantly lower cell migration and invasion ability in the sh-MCM3 group than in sh-Ctrl group (*P*<0.05, Figure 9B,C). To determine the effect of MCM3 knockdown on tumorigenesis, COLO205 cells transfected with sh-MCM3 and negative control cells were injected into the lateral abdomen of nude mice. The tumor growth (volume and weight) was dramatically inhibited in the sh-MCM3 group unlike in the control group (Figure 9D). IHC staining using PCNA as an indicator of tumor cell proliferation revealed the PCNA expression was substantially lower in the sh-MCM3 group compared with the control group (Figure 9E). Collectively, these results demonstrated that MCM3 regulated the progression of CRC cells.

## Discussion

Strict control of DNA replication is a prerequisite for normal cell division [41]. Therefore, deregulation of DNA replication leads to abnormal gene phenotypes that drive malignant transformation of normal cells [42,43]. Currently, chemotherapy and small-molecule inhibitors targeting key enzymes of the DNA replication process are used to treat tumors [44]. Given that these targeted drugs are non-specific, they affect the entire replication process of normal and tumor cells leading to concomitant death [45]. Prevailing evidence indicates that MCM family proteins act as helicases in the initial stage of DNA replication, and are key regulators of cell cycle checkpoints [46]. For instance, MCM2-7 complexes contribute to the initiation of cell cycle, and are therefore potential markers for tumor screening and treatment.

To the best of our knowledge, comprehensive bioinformatics analysis of the MCM family in CRC has not been performed. Our study is, therefore, the first to analyze mRNA expression profile of MCM family in CRC by integrating information from several large databases. We examined mRNA expression profiles of the MCM family in 20 common human tumors using the Oncomine database. The results showed that all MCM family members were up-regulated in CRC and these results were corroborated by immunohistochemistry analysis of colorectal cancer and normal tissues [47]. Immunostaining results from the HPA database demonstrated that the expression of the MCM family proteins in CRC was consistent with that of mRNA expression. Further, Kaplan–Meier analysis revealed that high expression of MCM3 was associated with poor DFS/PFS. No such association was found for other members of MCM family.

MCM3 is the catalytic core of the MCM2-7 complex which besides recognizing the origin of replication, generates ATP-dependent enzyme activity by interacting with proteins involved in the cell division cycle [48]. High expression of MCM3 has been reported in other types of neoplasia including papillary thyroid carcinoma [49], cervical squamous cell carcinoma [50], ameloblastoma [51] and malignant melanoma [52]. Consistently, our results reveal that MCM3 is highly expressed in CRC cell lines based on CCLE analysis and qPCR assay. These results show the potential of MCM3 to serve as a marker of tumor cell proliferation and a prognostic predictor.

Interestingly, Western blot analysis revealed that MCM3 was positively related to classic cyclins such as CDK2 and CDK4. This partially confirmed that MCM3 promotes DNA synthesis [53]. A study by Branzei et al. indicated that the MCM2–7 complex controls progression from G1 to S phase during replication by regulating DNA replication checkpoints [54]. Elsewhere, it was reported that deregulation of cell cycle promoted genomic instability and apoptosis leading tumorigenesis [55]. Consistent with these findings, analysis of the CancerSEA showed that MCM3 participated in the cell cycle, proliferation and metastasis. These functions were verified experimentally through *in vitro* and *in vivo* tests which showed that MCM3 deletion significantly suppressed the G1 phase and promoted apoptosis. Moreover, MCM3 knockdown dramatically inhibited COLO205 cell proliferation, migration and invasion both *in vitro* and *in vivo*. We therefore propose that MCM3 could be a promising biomarker of CRC.

Inevitably, our study has the following limitations. First, we only examined the relationship between expression of MCMs with overall prognosis, and not clinicopathological characteristics. Second, the results obtained from online databases were we verified using only one CRC cell line. Despite these shortcomings, we performed the first comprehensive expression profile analysis of the MCM family in CRC using multiple large tumor databases. Our results show that MCM3 is a potential therapeutic target in CRC.

## Abbreviations

CRC: colorectal cancer
FCM: flow cytometry
HPA: Human Protein Atlas
IHC: immunohistochemical
MCM: minichromosome maintenance

## References

[1] Bray F., Ferlay J., Soerjomataram I., Siegel R.L., Torre L.A. and Jemal A. (2018) Global cancer statistics 2018: GLOBOCAN estimates of incidence and mortality worldwide for 36 cancers in 185 countries. CA Cancer J. Clin. 68, 394–424 10.3322/caac.21492 30207593

[2] Siegel R.L., Miller K.D. and Jemal A. (2019) Cancer statistics, 2019. Ca A Cancer J. Clin. 69, 7–34 10.3322/caac.21551 30620402

[3] Sohrabi M., Zamani F., Ajdarkosh H., Rakhshani N., Ameli M., Mohamadnejad M. et al. (2014) Prevalence of colorectal polyps in a group of subjects at average-risk of colorectal cancer undergoing colonoscopic screening in Tehran, Iran between 2008 and 2013. Asian Pac J Cancer Prev. 15, 9773–9779 25520103 10.7314/apjcp.2014.15.22.9773

[4] Zhu J., Tan Z., Hollis-Hansen K., Zhang Y., Yu C. and Li Y. (2017) Epidemiological trends in colorectal cancer in China: An ecological study. Dig. Dis. Sci. 62, 235–243 10.1007/s10620-016-4362-4 27796769

[5] Brenner H., Kloor M. and Pox C.P. (2014) Colorectal cancer. Lancet 383, 1490–1502 10.1016/S0140-6736(13)61649-9 24225001

[6] Faubert B., Solmonson A. and DeBerardinis R.J. (2020) Metabolic reprogramming and cancer progression. Science 368, eaaw5473 10.1126/science.aaw5473 32273439 PMC7227780

[7] Boyer A.S., Walter D. and Sorensen C.S. (2016) DNA replication and cancer: From dysfunctional replication origin activities to therapeutic opportunities. Semin. Cancer Biol. 37-38, 16–25 10.1016/j.semcancer.2016.01.001 26805514

[8] Maine G.T., Sinha P. and Tye B.K. (1984) Mutants of S. cerevisiae defective in the maintenance of minichromosomes. Genetics 106, 365–385 6323245 10.1093/genetics/106.3.365 PMC1224244

[9] Moir D., Stewart S.E., Osmond B.C. and Botstein D. (1982) Cold-sensitive cell-division-cycle mutants of yeast: isolation, properties, and pseudoreversion studies. Genetics 100, 547–563 6749598 10.1093/genetics/100.4.547 PMC1201831

[10] Vijayraghavan S. and Schwacha A. (2012) The eukaryotic Mcm2-7 replicative helicase. Subcell. Biochem. 62, 113–134 10.1007/978-94-007-4572-8_7 22918583

[11] Johnson E.M., Kinoshita Y. and Daniel D.C. (2003) A new member of the MCM protein family encoded by the human MCM8 gene, located contrapodal to GCD10 at chromosome band 20p12.3-13. Nucleic Acids Res. 31, 2915–2925 10.1093/nar/gkg395 12771218 PMC156728

[12] Lutzmann M., Maiorano D. and Mechali M. (2005) Identification of full genes and proteins of MCM9, a novel, vertebrate-specific member of the MCM2-8 protein family. Gene 362, 51–56 10.1016/j.gene.2005.07.031 16226853

[13] Homesley L., Lei M., Kawasaki Y., Sawyer S., Christensen T. and Tye B.K. (2000) Mcm10 and the MCM2-7 complex interact to initiate DNA synthesis and to release replication factors from origins. Genes Dev. 14, 913–926 10783164 PMC316538

[14] Deegan T.D. and Diffley J.F. (2016) MCM: one ring to rule them all. Curr. Opin. Struct. Biol. 37, 145–151 10.1016/j.sbi.2016.01.014 26866665

[15] Snyder M., Huang X.Y. and Zhang J.J. (2009) The minichromosome maintenance proteins 2-7 (MCM2-7) are necessary for RNA polymerase II (Pol II)-mediated transcription. J. Biol. Chem. 284, 13466–13472 10.1074/jbc.M809471200 19318354 PMC2679446

[16] Wei Q., Li J., Liu T., Tong X. and Ye X. (2013) Phosphorylation of minichromosome maintenance protein 7 (MCM7) by cyclin/cyclin-dependent kinase affects its function in cell cycle regulation. J. Biol. Chem. 288, 19715–19725 10.1074/jbc.M112.449652 23720738 PMC3707676

[17] Chen Z.H., Yu Y.P., Michalopoulos G., Nelson J. and Luo J.H. (2015) The DNA replication licensing factor miniature chromosome maintenance 7 is essential for RNA splicing of epidermal growth factor receptor, c-Met, and platelet-derived growth factor receptor. J. Biol. Chem. 290, 1404–1411 10.1074/jbc.M114.622761 25425645 PMC4340387

[18] Zhai Y., Li N., Jiang H., Huang X., Gao N. and Tye B.K. (2017) Unique roles of the non-identical MCM subunits in DNA replication licensing. Mol. Cell 67, 168–179 10.1016/j.molcel.2017.06.016 28732205

[19] Bleichert F., Botchan M.R. and Berger J.M. (2017) Mechanisms for initiating cellular DNA replication. Science 355, eaah6317 10.1126/science.aah6317 28209641

[20] Natsume T., Nishimura K., Minocherhomji S., Bhowmick R., Hickson I.D. and Kanemaki M.T. (2017) Acute inactivation of the replicative helicase in human cells triggers MCM8-9-dependent DNA synthesis. Genes Dev. 31, 816–829 10.1101/gad.297663.117 28487407 PMC5435893

[21] Thu Y.M. and Bielinsky A.K. (2014) MCM10: one tool for all-Integrity, maintenance and damage control. Semin. Cell Dev. Biol. 30, 121–130 10.1016/j.semcdb.2014.03.017 24662891 PMC4043890

[22] Sagredo E.A., Sagredo A.I., Blanco A., De Santiago P.R., Rivas S., Assar R. et al. (2020) ADAR1 Transcriptome editing promotes breast cancer progression through the regulation of cell cycle and DNA damage response. Biochim. Biophys. Acta Mol. Cell Res. 118716 10.1016/j.bbamcr.2020.118716 32275931

[23] Wojnar A., Pula B., Piotrowska A., Jethon A., Kujawa K., Kobierzycki C. et al. (2011) Correlation of intensity of MT-I/II expression with Ki-67 and MCM-2 proteins in invasive ductal breast carcinoma. Anticancer Res. 31, 3027–3033 21868554

[24] Liao X., Liu X., Yang C., Wang X., Yu T., Han C. et al. (2018) Distinct Diagnostic and Prognostic Values of Minichromosome Maintenance Gene Expression in Patients with Hepatocellular Carcinoma. J. Cancer 9, 2357–2373 10.7150/jca.25221 30026832 PMC6036720

[25] Zhong H., Chen B., Neves H., Xing J., Ye Y., Lin Y. et al. (2017) Expression of minichromosome maintenance genes in renal cell carcinoma. Cancer Manag. Res. 9, 637–647 10.2147/CMAR.S146528 29180899 PMC5697450

[26] Pillaire M.J., Selves J., Gordien K., Gourraud P.A., Gentil C., Danjoux M. et al. (2010) A ‘DNA replication’ signature of progression and negative outcome in colorectal cancer. Oncogene 29, 876–887 10.1038/onc.2009.378 19901968

[27] Hua C., Zhao G., Li Y. and Bie L. (2014) Minichromosome Maintenance (MCM) family as potential diagnostic and prognostic tumor markers for human gliomas. BMC Cancer 14, 526 10.1186/1471-2407-14-526 25046975 PMC4223428

[28] Wu W., Wang X., Shan C., Li Y. and Li F. (2018) Minichromosome maintenance protein 2 correlates with the malignant status and regulates proliferation and cell cycle in lung squamous cell carcinoma. Onco. Targets Ther. 11, 5025–5034 10.2147/OTT.S169002 30174440 PMC6109654

[29] Wang D., Li Q., Li Y. and Wang H. (2018) The role of MCM5 expression in cervical cancer: Correlation with progression and prognosis. Biomed. Pharmacother. 98, 165–172 10.1016/j.biopha.2017.12.006 29253764

[30] Byun W.S., Kim S., Shin Y.H., Kim W.K., Oh D.C. and Lee S.K. (2020) Antitumor Activity of Ohmyungsamycin A through the Regulation of the Skp2-p27 Axis and MCM4 in Human Colorectal Cancer Cells. J. Nat. Prod. 83, 118–126 10.1021/acs.jnatprod.9b00918 31894983

[31] Qu K., Wang Z., Fan H., Li J., Liu J., Li P. et al. (2017) MCM7 promotes cancer progression through cyclin D1-dependent signaling and serves as a prognostic marker for patients with hepatocellular carcinoma. Cell Death. Dis. 8, e2603 10.1038/cddis.2016.352 28182015 PMC5386449

[32] Goldberg Y., Halpern N., Hubert A., Adler S.N., Cohen S., Plesser-Duvdevani M. et al. (2015) Mutated MCM9 is associated with predisposition to hereditary mixed polyposis and colorectal cancer in addition to primary ovarian failure. Cancer Genet. 208, 621–624 10.1016/j.cancergen.2015.10.001 26806154

[33] Frigola J., Remus D., Mehanna A. and Diffley J.F. (2013) ATPase-dependent quality control of DNA replication origin licensing. Nature 495, 339–343 10.1038/nature11920 23474987 PMC4825857

[34] Valverde L.F., de Freitas R.D., Pereira T.A., de Resende M.F., Agra I.M.G., Dos Santos J.N. et al. (2018) MCM3: A novel proliferation marker in oral squamous cell carcinoma. Appl. Immunohistochem. Mol. Morphol. 26, 120–125 27258565 10.1097/PAI.0000000000000397

[35] Rhodes D.R., Kalyana-Sundaram S., Mahavisno V., Varambally R., Yu J., Briggs B.B. et al. (2007) Oncomine 3.0: genes, pathways, and networks in a collection of 18,000 cancer gene expression profiles. Neoplasia 9, 166–180 10.1593/neo.07112 17356713 PMC1813932

[36] Ponten F., Jirstrom K. and Uhlen M. (2008) The Human Protein Atlas–a tool for pathology. J. Pathol. 216, 387–393 10.1002/path.2440 18853439

[37] Cerami E., Gao J., Dogrusoz U., Gross B.E., Sumer S.O., Aksoy B.A. et al. (2012) The cBio cancer genomics portal: an open platform for exploring multidimensional cancer genomics data. Cancer Discov. 2, 401–404 10.1158/2159-8290.CD-12-0095 22588877 PMC3956037

[38] Gao J., Aksoy B.A., Dogrusoz U., Dresdner G., Gross B., Sumer S.O. et al. (2013) Integrative analysis of complex cancer genomics and clinical profiles using the cBioPortal. Sci. Signal 6, pl1 10.1126/scisignal.2004088 23550210 PMC4160307

[39] Yuan H., Yan M., Zhang G., Liu W., Deng C., Liao G. et al. (2019) CancerSEA: a cancer single-cell state atlas. Nucleic Acids Res. 47, D900–D908 10.1093/nar/gky939 30329142 PMC6324047

[40] Barretina J., Caponigro G., Stransky N., Venkatesan K., Margolin A.A., Kim S. et al. (2012) The cancer cell line encyclopedia enables predictive modelling of anticancer drug sensitivity. Nature 483, 603–607 10.1038/nature11003 22460905 PMC3320027

[41] van Diest P.J., Brugal G. and Baak J.P. (1998) Proliferation markers in tumours: interpretation and clinical value. J. Clin. Pathol. 51, 716–724 10.1136/jcp.51.10.716 10023332 PMC500923

[42] Ichim C.V. and Wells R.A. (2006) First among equals: the cancer cell hierarchy. Leuk. Lymphoma 47, 2017–2027 10.1080/10428190600733325 17071472

[43] Swanton C. (2012) Intratumor heterogeneity: evolution through space and time. Cancer Res. 72, 4875–4882 10.1158/0008-5472.CAN-12-2217 23002210 PMC3712191

[44] Pogorelcnik B., Perdih A. and Solmajer T. (2013) Recent developments of DNA poisons–human DNA topoisomerase IIalpha inhibitors–as anticancer agents. Curr. Pharm. Des. 19, 2474–2488 10.2174/1381612811319130016 23363399

[45] Mazevet M., Moulin M., Llach-Martinez A., Chargari C., Deutsch E., Gomez A.M. et al. (2013) Complications of chemotherapy, a basic science update. Presse Med. 42, e352–e361 10.1016/j.lpm.2013.06.011 23972551

[46] Yu S., Wang G., Shi Y., Xu H., Zheng Y. and Chen Y. (2020) MCMs in cancer: prognostic potential and mechanisms. Anal Cell Pathol. (Amst.) 2020, 3750294 32089988 10.1155/2020/3750294 PMC7023756

[47] Palmqvist R., Oberg A., Bergstrom C., Rutegard J.N., Zackrisson B. and Stenling R. (1998) Systematic heterogeneity and prognostic significance of cell proliferation in colorectal cancer. Br. J. Cancer 77, 917–925 10.1038/bjc.1998.152 9528835 PMC2150107

[48] Bochman M.L. and Schwacha A. (2009) The MCM complex: unwinding the mechanism of a replicative helicase. Microbiol. Mol. Biol. Rev. 73, 652–683 10.1128/MMBR.00019-09 19946136 PMC2786579

[49] Lee Y.S., Ha S.A., Kim H.J., Shin S.M., Kim H.K., Kim S. et al. (2010) Minichromosome maintenance protein 3 is a candidate proliferation marker in papillary thyroid carcinoma. Exp. Mol. Pathol. 88, 138–142 10.1016/j.yexmp.2009.09.015 19818763

[50] Gan N., Du Y., Zhang W. and Zhou J. (2010) Increase of MCM3 and MCM4 expression in cervical squamous cell carcinomas. Eur. J. Gynaecol. Oncol. 31, 291–294 21077471

[51] Carreon-Burciaga R.G., Gonzalez-Gonzalez R., Molina-Frechero N. and Bologna-Molina R. (2015) Immunoexpression of Ki-67, MCM2, and MCM3 in Ameloblastoma and Ameloblastic Carcinoma and Their Correlations with Clinical and Histopathological Patterns. Dis. Markers 2015, 683087 10.1155/2015/683087 26823641 PMC4707386

[52] Nodin B., Fridberg M., Jonsson L., Bergman J., Uhlen M. and Jirstrom K. (2012) High MCM3 expression is an independent biomarker of poor prognosis and correlates with reduced RBM3 expression in a prospective cohort of malignant melanoma. Diagn. Pathol. 7, 82 10.1186/1746-1596-7-82 22805320 PMC3433373

[53] Maiorano D., Lutzmann M. and Mechali M. (2006) MCM proteins and DNA replication. Curr. Opin. Cell Biol. 18, 130–136 10.1016/j.ceb.2006.02.006 16495042

[54] Branzei D. and Foiani M. (2006) The Rad53 signal transduction pathway: Replication fork stabilization, DNA repair, and adaptation. Exp. Cell Res. 312, 2654–2659 10.1016/j.yexcr.2006.06.012 16859682

[55] Cho Y.J. and Liang P. (2011) S-phase-coupled apoptosis in tumor suppression. Cell. Mol. Life Sci. 68, 1883–1896 10.1007/s00018-011-0666-x 21437646 PMC11114674

